# Recent Development and Perspectives of Optimization Design Methods for Piezoelectric Ultrasonic Transducers

**DOI:** 10.3390/mi12070779

**Published:** 2021-06-30

**Authors:** Dongdong Chen, Linwei Wang, Xingjun Luo, Chunlong Fei, Di Li, Guangbao Shan, Yintang Yang

**Affiliations:** School of Microelectronics, Xidian University, Xi’an 710071, China; ddchen@xidian.edu.cn (D.C.); lwwang-1@stu.xidian.edu.cn (L.W.); xjluo_xidian@163.com (X.L.); clfei@xidian.edu.cn (C.F.); ytyang@xidian.edu.cn (Y.Y.)

**Keywords:** piezoelectric ultrasonic transducer, optimization design, finite element model, data-driven model, intelligent optimization algorithm

## Abstract

A piezoelectric ultrasonic transducer (PUT) is widely used in nondestructive testing, medical imaging, and particle manipulation, etc., and the performance of the PUT determines its functional performance and effectiveness in these applications. The optimization design method of a PUT is very important for the fabrication of a high-performance PUT. In this paper, traditional and efficient optimization design methods for a PUT are presented. The traditional optimization design methods are mainly based on an analytical model, an equivalent circuit model, or a finite element model and the design parameters are adjusted by a trial-and-error method, which relies on the experience of experts and has a relatively low efficiency. Recently, by combining intelligent optimization algorithms, efficient optimization design methods for a PUT have been developed based on a traditional model or a data-driven model, which can effectively improve the design efficiency of a PUT and reduce its development cycle and cost. The advantages and disadvantages of the presented methods are compared and discussed. Finally, the optimization design methods for PUT are concluded, and their future perspectives are discussed.

## 1. Introduction

Sound waves are a form of energy transmission in the mechanical vibration state of an object. Ultrasound is a type of sound wave with a vibration frequency higher than 20 kHz, which cannot be heard by humans [1]. A piezoelectric ultrasonic transducer (PUT) is a device for achieving mutual conversion of mechanical energy and electrical energy [2].

Due to the advantages of high safety and low cost, a PUT has been widely used as the core device for non-destructive testing (NDT), medical imaging, particle manipulation, and flow measurement [3,4,5,6]. In NDT, ultrasonic detection and the guided-wave structural health monitoring (SHM) method are combined to detect the damage of wind turbine blades, and the defect position can be precisely determined [7]. The annular PUT array printed by three-dimensional (3D) printing technology has adjustable focal domain and resolution and has been successfully used for NDT [8]. In medical imaging, the piezoelectric performance of a PUT is improved by polarizing to achieve high imaging resolution [9]. In addition, the inaccuracy of transcranial imaging caused by phase aberration can be eliminated by using ultrasonic adaptive beam forming to make ultrasonic imaging more accurate [10]. An acoustic radiation force optical coherence tomography system, using integrated micro ultrasound and optical coherence tomography, can map the correlated elasticity of vascular tissue to aid medical diagnosis [11]. In particle manipulation, the accurate control of ultrasound is extremely important for non-contact manipulation of biologics and bioanalysis [12]. Single-beam acoustic tweezers with pressure-focusing technology can be used as the manipulators in a wide range of biomedical and chemical sciences [13]. In addition, a bending PUT has been widely used for flow measurement under atmospheric pressure conditions [14]. A flexible piezocomposite ultrasound transducer can continuously measure blood pressure by tracking the ultrasonic motion of the vessel wall, and it can be used for non-invasive, non-obstructive, calibration-free blood pressure and blood flow monitoring [15]. Therefore, a PUT is the key component in specific ultrasonic application systems, and its performance mainly affects the performance and effectiveness of these systems.

**Figure 1:**
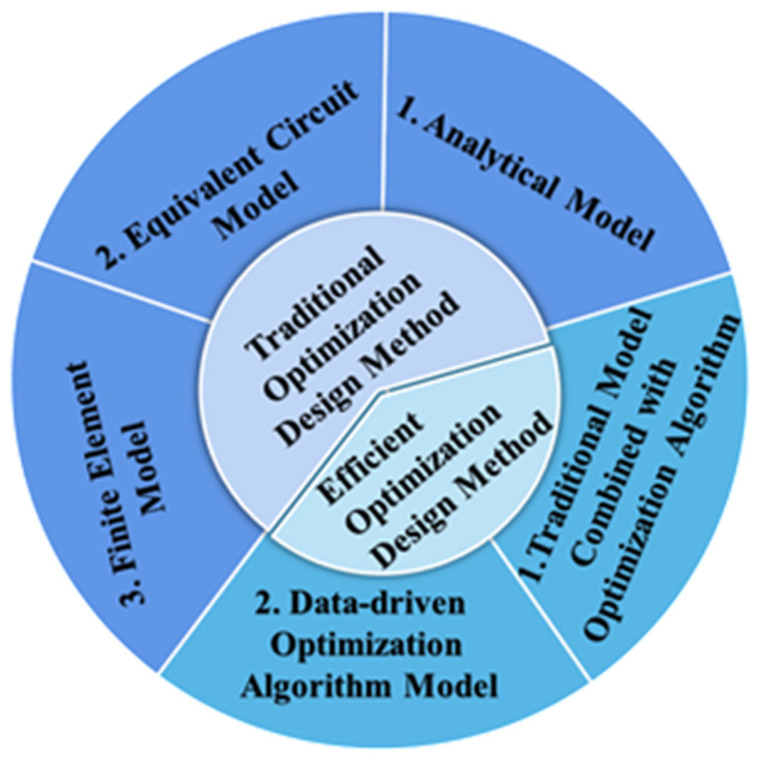
Optimization design methods for a piezoelectric ultrasonic transducer.

In order to fabricate a high-performance PUT, an effective optimization design method is essential and critical. In this paper, recent developments of optimization design methods for a PUT are systemically reviewed, as shown in Figure 1. The optimization design methods for a PUT include the traditional and efficient optimization design methods. The traditional optimization design methods are based on an analytical model, an equivalent circuit model (ECM), or a finite element model (FEM) and the design parameters are adjusted by a trial-and-error method. The efficient optimization methods are proposed based on a traditional model or a data-driven model, and intelligent optimization algorithms are utilized to optimize the design parameters of the PUT. The advantages and disadvantages of these optimization design methods for a PUT are systemically compared and discussed. Finally, future perspectives of the optimization design methods for a PUT are presented. 

## 2. Traditional Optimization Design Methods for a PUT

Generally, traditional optimization design methods for a PUT are mainly based on an analytical model, an ECM, or a FEM to determine the design parameters. An analytical model is derived from a wave equation, and an ECM is established by treating the PUT as a two-port device. A FEM is established based on the physical equations and its geometrical structure, which can accurately simulate the characteristics of a PUT.

### 2.1. Analytical Model

**Figure 2:**
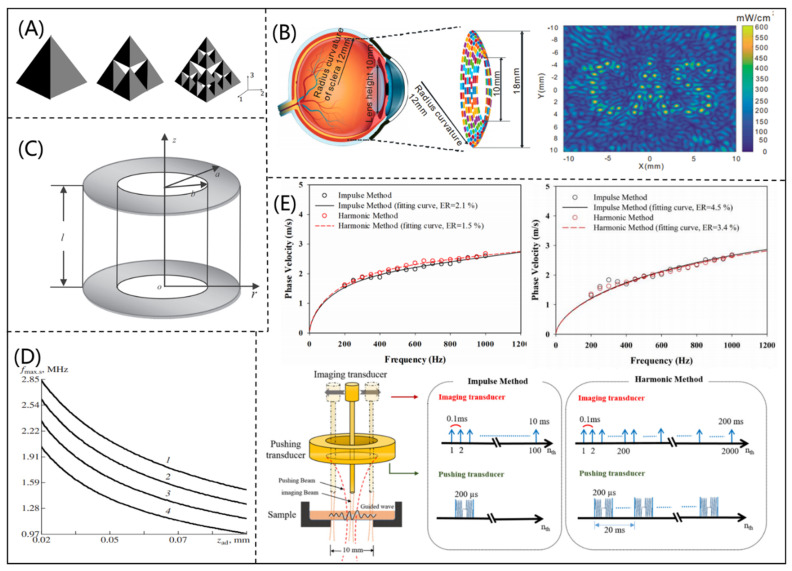
(**A**) 3D fractal structure for a piezoelectric ultrasonic transducer (reproduced from [16]); (**B**) simulation diagram of a racing array transducer applied to ultrasonic stimulation and the CAS pattern in ultrasonic field (reproduced from [17]); (**C**) schematic diagram of a piezoelectric tube (reproduced from [18]); (**D**) high-frequency piezoelectric ultrasonic transducer structure diagram and performance test diagram (reproduced from [19]); (**E**) porcine corneal and rabbit carotid artery phase velocity fitted lines, high-frequency ultrasonic microelastic imaging system (reproduced from [21]).

An analytical model is a relatively simple model that provides the analytical solution of the wave equation, which can describe the characteristics of ultrasonic propagation. Therefore, due to its simplicity of calculations and derivation of wave transport, an analytical model can be used to design a PUT. Because the regular geometry of a PUT limits its working bandwidth, Canning et al. [16] proposed a new three-dimensional (3D) fractal mathematical model for a PUT by studying the lattice structure of Sierpinski TETRIX, as shown in Figure 2A. The working bandwidth and amplitude of the PUT designed by the proposed model could be effectively improved. To improve the development of ultrasonic retinal stimulation, Yu et al. [17] proposed a racing array transducer with a contact lens shape by using the discrete Rayleigh–Sommerfeld method. According to the analytical model, the “CAS” pattern was constructed in the ultrasonic field (shown in Figure 2B), which could achieve a stimulus resolution of about 0.6 mm. Using three-dimensional motion equations and an electrostatic charge equation, Zhang et al. [18] deduced the analytical resonance frequency equation of coupled longitudinal and radial vibrations for a longitudinally polarized piezoelectric tube, as shown in Figure 2C. The calculated resonant frequency showed good agreement with the simulated resonant frequency by a FEM, which proved the reliability of the analytical model. In addition, Danilov et al. [19] deduced the center frequency (CF) estimation formula for a PUT and found that adding an acoustic impedance matching layer could significantly increase the working signal frequency, as shown in Figure 2D. Gorostiaga et al. [20] derived an analytical expression for ultrasonic receivers at an optimal electrical load, which could minimize energy loss. Combining lamb wave modeling with ultrasonic microelastic imaging techniques, Shih et al. [21] developed a microelastic imaging system that could improve spatial resolution and measured the shear viscoelasticity of thin layers by using this imaging system, as shown in Figure 2E.

An analytical model is simple and can be easily used in the design of a PUT. However, there are assumptions and simplifications in an analytical model, and therefore its accuracy is low, which leads to low accuracy for designing a PUT. In traditional optimization design methods, the accuracy of a model determines the efficiency of designing a PUT, therefore, it is necessary to improve the accuracy of models.

### 2.2. Equivalent Circuit Model

An ECM is a type of resistance matching network, which takes different layers, such as piezoelectric, backing, and matching layers, as loads on the transmission line. In an ECM, the acoustic impedance and electrical resistance matching networks of a PUT can be studied separately, which makes the model more accurate [22]. In addition, the complexity of an ECM is significantly simplified. However, an ECM cannot consider all the design parameters of a PUT and has some limitations [23]. The Krimholtz, Leedom and Mattaei (KLM) model and the Mason model are general ECMs and have been widely used for designing a PUT [24,25].

**Figure 3:**
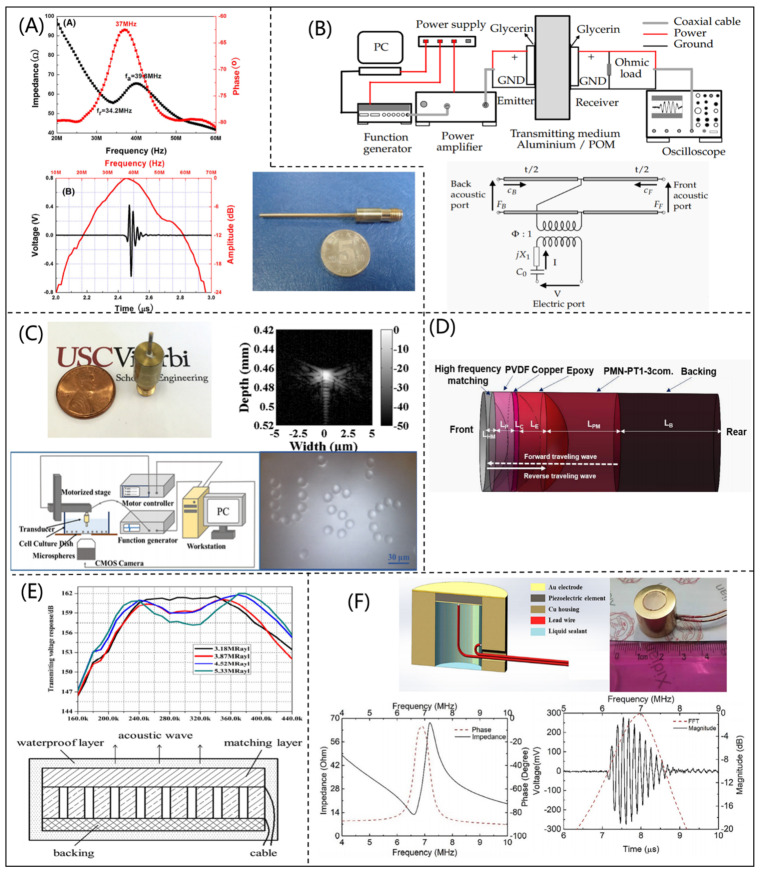
(**A**) Electrical impedance amplitude and phase variation with frequency, pulse-echo waveform, and spectrum of needle piezoelectric ultrasonic transducer (reproduced from [26]); (**B**) ultrasonic power transfer experimental setup diagram and KLM equivalent circuit diagram (reproduced from [26]); (**C**) needle-type piezoelectric ultrasonic transducer, 4 um tungsten wire imaging schematic, and acoustic tweezers manipulating particles to form USC patterns (reproduced from [27]); (**D**) schematic diagram of a dual-frequency confocal transducer (reproduced from [28]); (**E**) 1-3 piezoelectric composite ultrasound transducer structure, emission voltage response, and simulated impedance (reproduced from [29]); (**F**) BDF-PT ultrasonic transducer (reproduced from [30]).

According to the KLM model, Ou-Yang et al. [26] designed and fabricated a 37 MHz high-frequency needle PUT, which was made of KNNS-BNKZ material, as shown in Figure 3A. Compared with PUTs made with other materials, the fabricated PUT had high electromechanical coupling coefficients and low insertion loss. Additionally, Kar et al. [27] constructed a contactless ultrasonic power transmission system by using the KLM model, as shown in Figure 3B. This system had metal shielding effect, which could effectively reduce the energy transmission loss. The PizeoCAD software (Sonic Concepts, Woodinville, WA), which is based on the KLM model, has been widely used for designing a PUT. Fei et al. [28] designed and manufactured an ultra-high frequency PUT with a CF higher than 300 MHz by using PizeoCAD software. The ultra-high frequency PUT could be used as the acoustic tweezer to precisely manipulate a particle, as shown in Figure 3C. This needle-type PUT could manipulate a single microsphere as small as 3 μm, and therefore has great potential in biomedical applications. Deng et al. [29] developed a PUT with dual frequency (5 MHz transmission and 30 MW reception) for microvascular imaging based on the KLM model. As shown in Figure 3D, the PUT included double layers of piezoelectric materials (PMN-PT single crystal and PVDF). The fabricated PUT had a bandwidth of 79%, which meant it had excellent performance. In the optimization design of a piezocomposite ultrasonic transducer, Chao et al. [30] designed a 1-3 piezocomposite PUT with a matching layer by using an ECM and acoustic theory, as shown in Figure 3E. The errors between theoretical and experimental results of the 1-3 piezocomposite PUT were less than 5.3%. In order to achieve NDT at high temperatures, Sun et al. [31] designed a PUT with a CF of 7 MHz based on PiezoCAD software, as shown in Figure 3F. In addition, Quan et al. [32] designed a Put with high frequency using PiezoCAD software. The fabricated PUT had a CF of higher than 80 MHz and a −6 dB bandwidth of 52%, which had excellent resolution for ultrasonic imaging.

**Figure 4:**
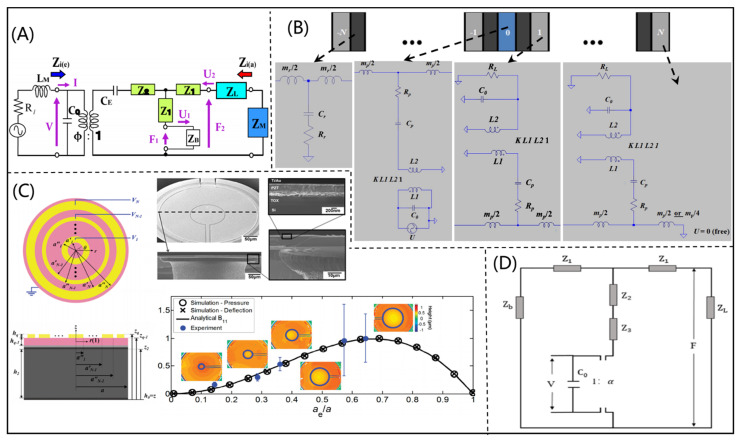
(**A**) Mason’s equivalent circuit for an ultrasonic wireless power transmission system (reproduced from [33]); (**B**) 1D equivalent circuit for ultrasonic transducer array (reproduced from [33]); (**C**) simulation diagram of a circular ultrasonic transducer (reproduced from [34]); (**D**) Mason model equivalent circuit for a 2-2 piezocomposite ultrasonic transducer (reproduced from [35]).

The Mason model is another common ECM for the design of a PUT. According to the Mason model (shown in Figure 4A), Hori [33] et al. determined the design parameters of a PUT, and the manufactured PUT had high transmission efficiency. For medical imaging and NDT, Bybi et al. [34] proposed a one-dimensional ECM for ultrasonic transducer array based on the Mason model (shown in Figure 4B), which could be used to solve the phenomenon of interference. Smyth et al. [35] derived the Mason model for a circular splint PUT (as shown in Figure 4C) at 31 modes. The practicability of the derived model was verified by electro impedance measurement experiments. The proposed model could be widely used in the field of medical ultrasound applications and NDT. To better match the impedance of piezoelectric material with the load medium, Hou et al. [36] designed a 2-2 piezocomposite ultrasonic transducer based on the Mason model (shown in Figure 4D). Due to the ideal electrical properties of piezocomposites, the bandwidth of the 2-2 piezocomposite ultrasonic transducer was increased from 21.28% to 35.54%. 

An ECM can quickly simulate the electrical properties of a PUT on the basis of given materials and parameters. With the advantages of simple calculations and accuracy, ECMs has been widely used in the optimization design of PUTs. However, the design parameters considered in an ECM are finite, which cannot effectively and comprehensively design a PUT. 

### 2.3. Finite Element Model

A FEM is a common modeling and simulation method for engineering applications, and it has been used to simulate the acoustic, electric and other multi-physical fields of a PUT. A FEM does not have the limitations of an analytical model and an ECM, and it has been utilized to design various PUTs.

**Figure 5:**
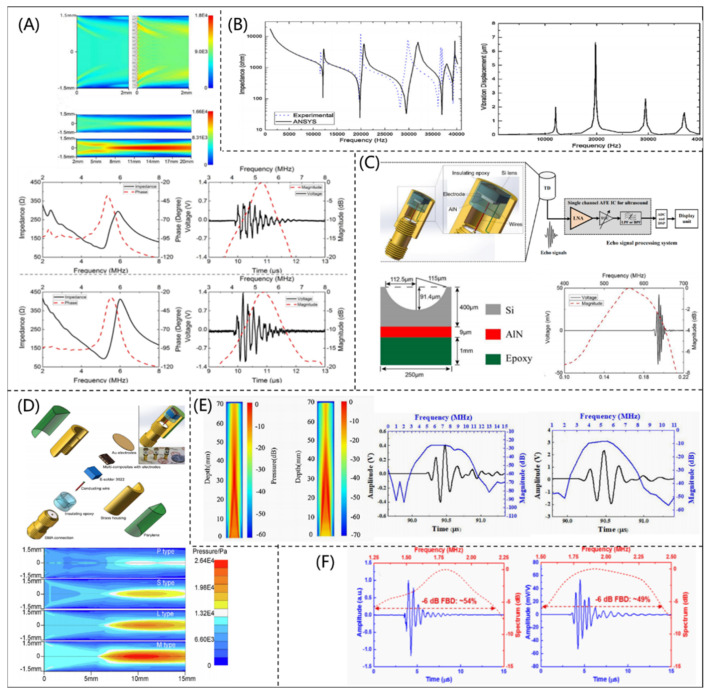
(**A**) Radiation pattern diagrams of graded ultrasonic transducer, impedance, and pulse echo diagrams of conventional and graded ultrasonic transducers (reproduced from [37]); (**B**) ANSYS (Ansys Inc., Canonsburg, PA, USA) simulation results of impedance and vibration displacement (reproduced from [37]); (**C**) simulated pulse-echo waveform, spectrum, and schematic diagram of a focused high-frequency piezoelectric ultrasonic transducer (reproduced from [38]); (**D**) physical diagrams of different types of piezoelectric ultrasonic transducers and simulated radiation patterns (reproduced from [39]); (**E**) simulated acoustic field, the impulse echo response of a conventional ultrasonic transducer, and a double piezoelectric layer ultrasonic transducer with PMNT + PZT (reproduced from [40]); (**F**) impulse response of a piezoelectric ultrasonic transducer obtained by the KLM model (reproduced from [41]).

On the basis of a vibration mode simulated by a FEM, Fei et al. [37] designed and fabricated a high sensitivity PUT with functional gradations (as shown in Figure 5A). Compared with a traditional PUT, the insertion losses of the fabricated PUT were significantly reduced by using the graded design. Li et al. [38] analyzed and optimized the geometrical parameters of a piezoelectric vibrator with flexural bending mode by using a coupled-field FEM. Higher electromechanical conversion efficiency and acoustic radiation efficiency could be achieved, as shown in Figure 5B. To realize an ultra-high frequency system, Li et al. [39] designed a tightly focused 500 MHz PUT by a FEM (shown in Figure 5C). In order to meet the requirement of a high-quality sensor, Lin et al. [40] proposed a 5 MHz piezocomposite ultrasonic transducer based on the finite element analysis software PZFlex (Weidlinger Associates, Cupertino, USA), as shown in Figure 5D, and found that the piezocomposite ultrasonic transducer had a wider bandwidth (40.6 %) and a higher peak voltage (18 mv) than the PUT fabricated by PZT. In addition, Liu et al. [41] designed a PUT with two piezoelectric layers (PMNT and PZT materials) by using PZFlex. The PUT had the highest performance when the thickness ratio of the two piezoelectric materials was 7.5:2.5 (as shown in Figure 5E). Similarly, Kim et al. [42] developed a flexible piezocomposite ultrasonic transducer combining PZT-5H and PDMS. The performance of the flexible piezocomposite ultrasonic transducer was simulated using ANSYS software (Ansys Inc., Canonsburg, PA, USA), as shown in Figure 5F. The result of the hydrophone test showed that the designed piezocomposite ultrasonic transducer had strong mechanical flexibility (bending radius lower than 5 mm) and high sensitivity. 

**Figure 6:**
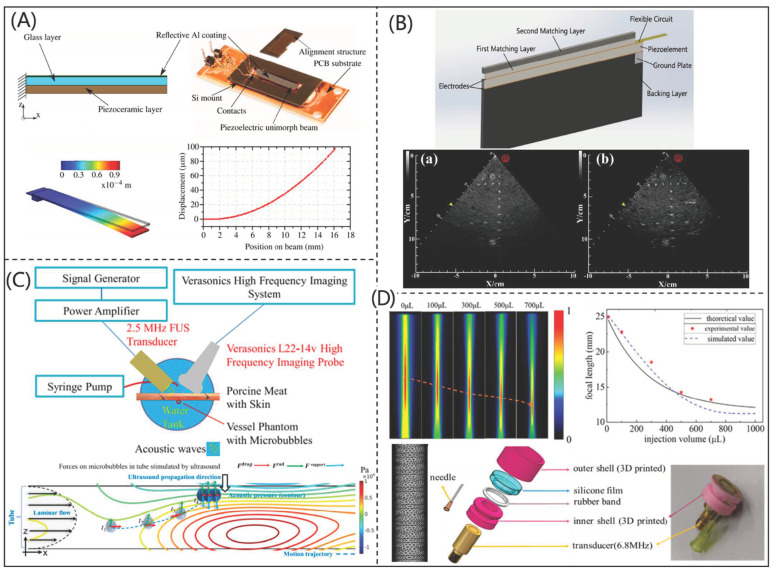
(**A**) Piezoelectric beam system, simulated single crystal sheet beam, and its bending profile (reproduced from [43]); (**B**) phased array ultrasonic transducer and the imaging results (reproduced from [43]); (**C**) schematic of a manipulated microbubble device (reproduced from [44]); (**D**) photograph and performance of piezoelectric ultrasonic transducer with liquid lens (reproduced from [45]).

COMSOL Multiphysics (COMSOL Inc., Burlington, MA, USA) is an effective multi-physical finite element simulation software, which has been widely used in the simulation and design of PUTs. Bruno et al. [43] investigated the performance of a typical bending actuator fabricated with PZT ceramics and PMN-PT material by using COMSOL software, as shown in Figure 6A. In addition, Zhang et al. [44] designed and fabricated a medical phased array ultrasonic transducer (as shown in Figure 6B) based on COMSOL software. The fabricated phased array ultrasonic transducer had a CF and –6 dB bandwidth of 3.0 MHz and 89.9%, respectively. Its axial and transverse resolutions were 0.5 and 1.8 mm, respectively, which showed excellent performance for biomedical imaging. With the help of COMSOL software, Peng et al. [45] proposed an ultrafast ultrasound imaging system for capturing mobile microbubbles, as shown in Figure 6C, which could be used to control drugs in an accurate location for clinical use. Similarly, Li et al. [46] designed and fabricated an acoustic liquid lens using COMSOL software for a PUT and improved the imaging performance, as shown in Figure 6D. The results showed that the acoustic liquid lens combined with a 6.8 MHz PUT had a tunable focus, which made accurate imaging in different conditions possible. 

A FEM can comprehensively and accurately simulate the multi-physical fields of a PUT, which is useful for its optimization design and fabrication. However, a FEM has a large amount of computations and long calculation time, which is not beneficial for decreasing the research and development cycle and cost of a PUT.

## 3. Efficient Optimization Design Methods for a PUT

Traditional optimization design methods for a PUT are developed based on an analytical model, an ECM, or a FEM, and the design parameters are determined by using a trial-and-error method, which is inefficient and depends on the knowledge of experts. Efficient and intelligent optimization design methods for a PUT are proposed and developed based on intelligent optimization algorithms. In the efficient optimization design methods, a traditional model or a data-driven model is used to describe the relationships among design and performance parameters of a PUT. 

### 3.1. Optimization Design Methods Based on Traditional Models

A simple and efficient optimization design method for a PUT can be developed by combining the traditional models and intelligent optimization algorithms. The traditional models can characterize the effects of design parameters on the performance of a PUT, and the intelligent optimization algorithms are utilized to optimize the design parameters. 

**Figure 7:**
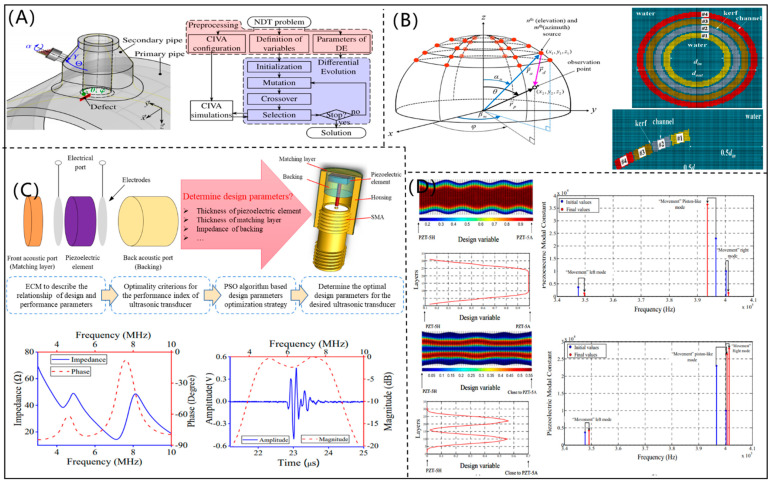
(**A**) Evolutionary algorithm-based optimization design method for a piezoelectric ultrasonic transducer (reproduced from [47]); (**B**) mathematical model for a concave annular high intensity focused ultrasonic transducer and its finite element model (reproduced from [47]); (**C**) optimization design for a piezoelectric ultrasonic transducer using the particle swarm optimization algorithm (reproduced from [48]); (**D**) functionally graded piezoelectric ultrasonic transducer optimized by using the topological optimization algorithm (reproduced from [49]).

Using CIVA software, Puel et al. [47] constructed a multiobjective optimization function for the optimization design of a PUT, and the evolutionary algorithm was used to optimize the design parameters, as shown in Figure 7A. According to the required performance of detection, the CF, size, and kerf of element could be optimized by the developed method. According to the principle of sound field, Choi et al. [48] proposed a mathematical model to describe the acoustic field distribution, as shown in Figure 7B. Then, the design parameters of a concave annular high intensity focused ultrasonic transducer could be optimized by using a nonlinear programming algorithm, which was beneficial for fabricating a high-intensity focused ultrasonic transducer. By combining an ECM and the particle swarm optimization (PSO) algorithm, Chen et al. [49] developed an optimization design method for PUT (as shown in Figure 7C). According to the optimized design parameters, the fabricated PUT had a CF of 6.3 MHz and a −6 dB bandwidth of 68.25%. With the combination of topology optimization and a FEM, Rubio et al. [50] designed a functional gradient PUT, (Figure 7D). The design parameters of the functional gradient PUT were optimized by using a topological optimization algorithm, which effectively improved the performance of the PUT. 

Due to the simple and fast calculations of an analytical model and an ECM, they can be easily combined with an intelligent optimization algorithm to design a PUT, but only several design parameters can be optimized due to the limitations of the analytical model and ECM. Although a FEM can be combined with an intelligent optimization algorithm to design a PUT, the design efficiency is very low because a FEM is very time-consuming. 

### 3.2. Optimization Design Methods Based on a Data-Driven Model

Due to the limitation of traditional models, the optimization design of a PUT cannot be designed in an efficient or comprehensive way. A data-driven model is established based on data and can be used to accurately and quickly describe the relationships among design and performance parameters, which can effectively improve the optimization design efficiency for a PUT.

**Figure 8:**
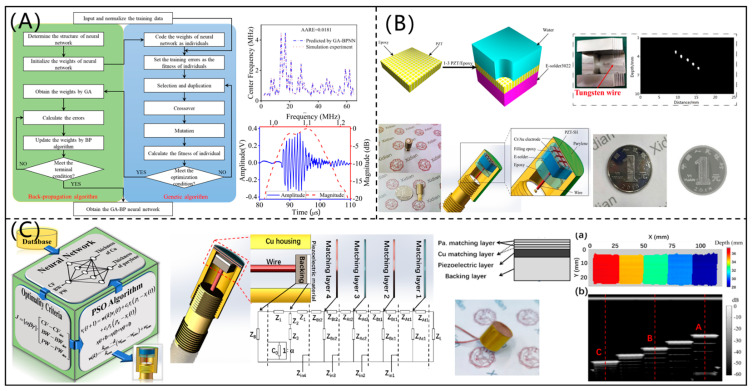
(**A**) Optimization design method for a high-performance transmitting piezoelectric ultrasonic transducer (reproduced from [51]); (**B**) designed and fabricated 1-3 piezocomposite ultrasonic transducer for ultrasonic imaging (reproduced from [51]); (**C**) optimization design method for a piezoelectric ultrasonic transducer with multi-match layers and testing results (reproduced from [52]).

On the basis of a genetic algorithm-based back-propagation neural network (GABPNN) model and the PSO algorithm, Chen et al. [51] proposed an optimization design method for a high-performance transmitting PUT (shown in Figure 8A). The GABPNN models were trained using the data simulated by PiezoCAD software to describe the relationships among the design and performance parameters of the PUT, and the PSO algorithm was adopted to determine the design parameters. The CF and insertion loss of the designed PUT were 1.07 MHz and 7.2 dB, respectively, which had excellent transmitting performance. In addition, Chen et al. [52] designed a 1-3 piezocomposite ultrasonic transducer for imaging application based on a data-driven model and modified PSO algorithm. As shown in Figure 8B, the ultrasonic images showed that the designed 1-3 piezocomposite ultrasonic transducer had high resolution and good signal-to-noise ratio. On the basis of intelligent optimization algorithms, Li et al. [53] designed a PUT with multi-match layers, as shown in Figure 8C. The CF, –6 dB bandwidth, and pulse width of the designed PUT were 5.672 MHz, 50.08%, and 0.295 us, respectively, which were significantly better than those of the PUTs without a matching layer. Additionally, the testing error of five-step block thickness was less than 1.0%.

**Figure 9:**
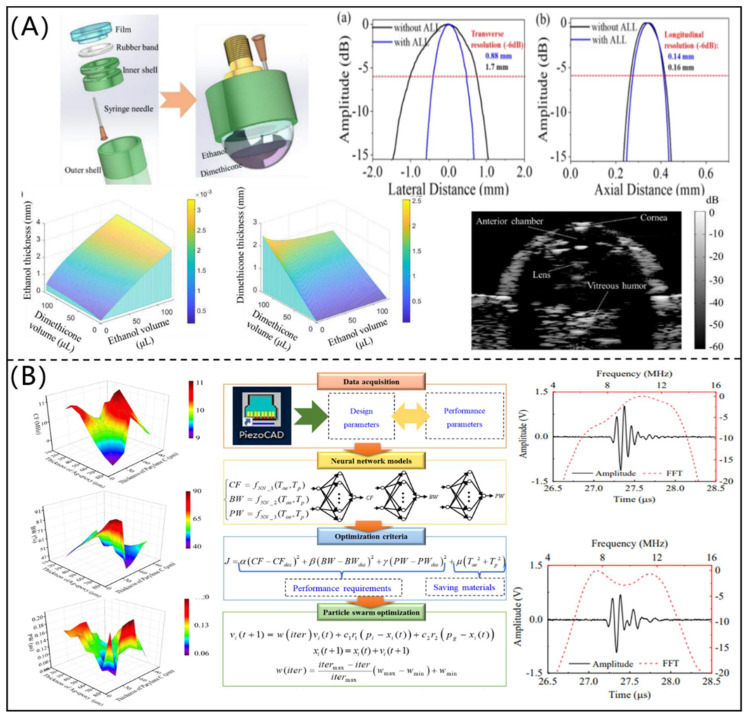
(**A**) Acoustic liquid lens and ultrasonic imaging results of porcine eye (reproduced from [54]); (**B**) Effect of matching layers on the performance of a piezoelectric ultrasonic transducer, and the intelligent optimization design of piezoelectric ultrasonic transducer with two matching layers (reproduced from [54]).

In order to effectively and flexibly control the acoustic field, Li et al. [54] proposed an efficient optimization design method for an acoustic liquid lens (as shown in Figure 9A) under the PSO framework. Tungsten wires and porcine eye were imaged by the 6 MHz PUT with an optimized acoustic liquid lens, and the ultrasonic imaging quality was obviously improved, which verified the reliability and effectiveness of the developed method. As shown in Figure 9B, Sun et al. [55] proposed an intelligent optimization design method for matching layers of a PUT. The neural network models were trained to establish the mapping relationships among the thicknesses of matching layers and performance parameters of the PUT. An improved PSO algorithm was used to optimize the thicknesses of matching layers (Ag-epoxy and parylene C). It was found that the PUT with the optimized matching layers had a −6 dB bandwidth of 68.5% and pulse echo width of 0.123 us, which were superior to those of the PUT with matching layers determined by the traditional quarter-wavelength theory. 

In the efficient optimization design method, a data-driven model can be established to represent the relationships among the design parameters and the performance parameters of a PUT, and an intelligent optimization algorithm is used to determine the design parameters. This method is relatively efficient and can reduce the design cycle and cost for a PUT. However, a large amount of data should be obtained and accumulated to establish the data-driven model, and therefore the data acquisition is the key for this method. 

## 4. Comparison and Discussion

In the optimization design of a PUT, the predictive model is very important, which mainly determines the design accuracy. In addition, the optimization strategy also plays an important role in the optimization design of a PUT, which determines the design efficiency. The comparison of traditional and efficient optimization design methods for a PUT is presented in Table 1.

In traditional optimization design methods for a PUT, a traditional model is used as the predictive model, and the optimization strategy is the simple trial-and-error method, which relies on the experience of an expert. An analytical model and an ECM are relatively simple models. The accuracy of an ECM is relatively high, but the design parameters considered in an ECM are finite. Although a FEM is precise and comprehensive, it is not conducive to shorten the design cycle of a PUT, due to large amounts of calculations and storage.

In the efficient optimization method based on a traditional model, the traditional model is also used as the predictive model, but an intelligent optimization algorithm is utilized in the optimization strategy, which can effectively improve the design efficiency. However, due to the limitation of traditional models, this efficient optimization design method for a PUT also has several disadvantages which existed in the traditional optimization design methods. Compared with the traditional model-based optimization design methods, a data-driven model-based optimization design method for a PUT has high efficiency, high reliability, and a low design cycle. However, the establishment of a data-driven model requires a large amount of data. With the accumulation of data, the efficient optimization design method based on a data-driven model is an effective way for the design and fabrication of a PUT.

## 5. Conclusions and Perspectives

In this paper, traditional and intelligent optimization methods for a PUT are summarized and compared. The traditional optimization design methods are generally based on an analytical model, an ECM, or a FEM, and the design parameters of a PUT are determined by trial-and-error method, which is inefficient and depends on the knowledge of experts. In the efficient optimization design methods for a PUT, a traditional model or a data-driven model can be used as the predictive model, and an intelligent optimization algorithm is adopted to determine the design parameters, which can effectively improve the design efficiency. The efficient optimization design methods can make the design of a PUT more efficient and intelligent, however, a data-driven model is established based on a large amount of data. 

Compared with traditional optimization design methods, the design efficiency of an efficient optimization design method is greatly improved. With the development of artificial intelligence, the intellectualized design for a PUT is a promising trend. In the future, the development and perspectives for the optimization design method of a PUT should include the following:

(1) The predictive model is very important for the design accuracy of a PUT; therefore, a high-precision predictive model should be developed based on traditional models.
(2) A large amount of original data should be accumulated to establish the database, which can be used to train data-driven models.
(3) Efficient optimization design strategies or algorithms should be developed to further improve the efficiency of the optimization design for a PUT.
(4) Efficient optimization design software should be developed by integrating the predictive models and intelligent optimization algorithms.
(5) Most optimization design methods are developed for a low-frequency PUT [28,36,37,40,46], therefore, an efficient optimization design method for a high-frequency PUT should be proposed and developed in the future.

## Figures and Tables

**Table 1:** Comparison of traditional and efficient optimization design methods for piezoelectric ultrasonic transducers.

Methods		Advantages	Disadvantages
Traditional optimization design methods	Analytical model	Easy and rapid calculation, simple model	Low accuracy, relying on the experience of expert
	Equivalent circuit model	Easy and rapid calculation, simple and accurate model	Finite parameters considered in this model, relying on the experience of an expert
	Finite element model	High accuracy, comprehensive model	Large calculation and memory, relying on the experience of an expert, long design cycle
Efficient optimization design methods	Traditional model	Simple method, high efficiency, low design cycle	Limitation of traditional models
	Data-driven model	High efficiency, high reliability, low design cycle	Requiring large amount of data
